# Dicarbon­yl(η^5^-cyclo­penta­dien­yl)[2-(phenyl­sulfan­yl)eth­yl]iron(II)

**DOI:** 10.1107/S160053681101511X

**Published:** 2011-04-29

**Authors:** Dennis S. Mkhize, Vincent O. Nyamori, Muhammad D. Bala

**Affiliations:** a School of Chemistry, University of KwaZulu-Natal, Westville Campus, Private Bag X54001, Durban 4000, South Africa

## Abstract

The title compound, [Fe(C_5_H_5_)(C_8_H_9_S)(CO)_2_], is a three-legged piano-stool iron(II) complex that is characterized by a thio­ethyl-linked phenyl ring and a cyclo­penta­dienyl moiety that occupies the apical coordination site. The two aromatic rings are essentially planar with the same maximum deviation of 0.009 Å. The mean planes of the phenyl and cyclo­penta­dienyl rings bis­ect at an acute angle of 50.08°.

## Related literature

For general background and related synthesis, see: King & Bisnette (1965*a* ▶,*b* ▶); Theys *et al.* (2009 ▶); Nyamori *et al.* (2008 ▶). For related structures, see: O’Connor *et al.* (1987 ▶).
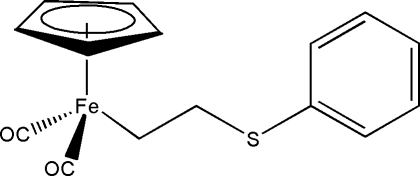


## Experimental

### 

#### Crystal data

[Fe(C_5_H_5_)(C_8_H_9_S)(CO)_2_]
*M* *_r_* = 314.17
Orthorhombic, 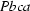

*a* = 10.3992 (5) Å
*b* = 7.6402 (4) Å
*c* = 35.4173 (16) Å
*V* = 2814.0 (2) Å^3^
*Z* = 8
Mo *K*α radiation
μ = 1.21 mm^−1^
*T* = 173 K
0.57 × 0.27 × 0.08 mm

#### Data collection

Bruker APEXII CCD area-detector diffractometer
Absorption correction: integration (*SADABS*; Bruker, 2009 ▶) *T*_min_ = 0.545, *T*_max_ = 0.909
20490 measured reflections
2752 independent reflections
2339 reflections with *I* > 2σ(*I*)
*R*_int_ = 0.068

#### Refinement

*R*[*F* ^2^ > 2σ(*F* ^2^)] = 0.075
*wR*(*F* ^2^) = 0.153
*S* = 1.26
2752 reflections
172 parameters
H-atom parameters constrained
Δρ_max_ = 0.49 e Å^−3^
Δρ_min_ = −0.75 e Å^−3^

### 

Data collection: *APEX2* (Bruker, 2009 ▶); cell refinement: *SAINT* (Bruker, 2009 ▶; data reduction: *SAINT*; program(s) used to solve structure: *SHELXS97* (Sheldrick, 2008 ▶); program(s) used to refine structure: *SHELXL97* (Sheldrick, 2008 ▶); molecular graphics: *ORTEP-3* (Farrugia, 1997 ▶); software used to prepare material for publication: *SHELXTL* (Sheldrick, 2008 ▶).

## Supplementary Material

Crystal structure: contains datablocks global, I. DOI: 10.1107/S160053681101511X/dn2676sup1.cif

Structure factors: contains datablocks I. DOI: 10.1107/S160053681101511X/dn2676Isup2.hkl

Additional supplementary materials:  crystallographic information; 3D view; checkCIF report

## Figures and Tables

**Fig. 1.**
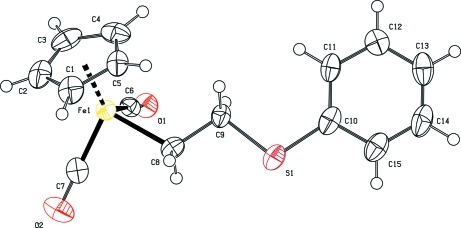
Molecular structure of the title compound with the atom numbering scheme. Displacement ellipsoids are drawn at the 50% probability level. H atoms are presented as small spheres of arbitrary radii.

## supplementary crystallographic information

### Comment

Organosulfur compounds of iron are valuable compounds that are used for the synthesis of organometallic catalysts and catalysts precursor salts. Amongst many applications this salts have been used for the cyclopropanation of alkenes (O'Connor *et al.*, 1987). The recent proliferation and interest in the chemistry of materials has also rekindled interest in Fe-based organometallics as catalysts for the synthesis of carbon nanotubes and nanomaterials in general (Nyamori *et al.*, 2008).

We are currently studying the application of (I) as a precursor catalyst in the synthesis of carbon nanomaterials. The presence of sulfur in this compound was believed to play the role of increasing the yield and affecting the morphology of the products obtained. In the title compound (I), the coordination of ligands around the Fe(II) ion is described as three-legged piano stool distorted octahedral geometry (Fig. 1). This is a typical geometry for complexes based on the dicarbonyl(η^5^-cyclopentadienyl)Fe(II) moiety (generally abbreviated as the Fp anion) and many compounds of iron with this geometry are well documented (Theys, *et al.*, 2009; O'Connor *et al.*, 1987).

### Experimental

A solution of cyclopentadienylirondicarbonyl dimer (1.501 g, 0.424 mmol) in dry tetrahydrofuran was treated with Na/Hg amalgam [sodium (0.2902 g, 12.70 mmol) and mercury (2.8 ml, 18.75 mmol)] and stirred at room temperature for two hours. The resulting yellowish-brown solution was transferred into another flask *via* a cannula and reacted with 2-chloroethyl phenyl sulfide (0.729 g, 4.242 mmol). The reaction mixture was allowed to stir for 16 h at room temperature. After concentrating the greyish brown mixture *in vacuo* it was extracted with dichloromethane (3 × 30 ml). Filtration of the extracted product through a celite pad afforded a clear brownish-orange filtrate which was further reduced *in vacuo*. The brown oil that resulted was purified by flash collumn chromatography on silica using hexane as mobile phase. A dark yellow band was collected and removal of volatiles afforded 0.8183 g of the final product as a yellow crystalline solid (yield, 61%).

^1^H NMR δ_H_ (400 MHz, CDCl_3_, p.p.m.): 1.58 (2*H*, t, *J* 8.56, CH_2_), 3.02 (2*H*, t, *J* 8.40, CH_2_), 4.68 (5*H*, s, Cp), 7.08 (1*H*, t, ArH), 7.20 (2*H*, m, *J*16.4, ArH_2_), 7.26 (2*H*, m, *J* 16.5, ArH_2_).

^13^C NMR (400 MHz, CDCl_3_, p.p.m.): 40.72, 84.03, 124.3, 127.6, 127.8, 136.1, 215.6.

IR (*ν*_CO_, cm^-1^): 1993, 1937, 1708.

### Refinement

All H-atoms were refined using a riding model, with C—H = 0.93 Å and *U_i_*_so_(H) = 1.2*U*_eq_(C) for aromatic and C—H = 0.97 Å and *U_i_*_so_(H) = 1.2*U*_eq_(C) for CH_2_.

### Crystal data

**Table:**

[Fe(C_5_H_5_)(C_8_H_9_S)(CO)_2_]	*F*(000) = 1296
*M**_r_* = 314.17	*D*_x_ = 1.483 Mg m^−^^3^
Orthorhombic, *P**b**c**a*	Mo *K*α radiation, λ = 0.71073 Å
Hall symbol: -P 2ac 2ab	Cell parameters from 6363 reflections
*a* = 10.3992 (5) Å	θ = 2.3–27.3°
*b* = 7.6402 (4) Å	µ = 1.21 mm^−^^1^
*c* = 35.4173 (16) Å	*T* = 173 K
*V* = 2814.0 (2) Å^3^	Plate, yellow
*Z* = 8	0.57 × 0.27 × 0.08 mm

### Data collection

**Table:**

Bruker APEXII CCD area-detector diffractometer	2752 independent reflections
Radiation source: fine-focus sealed tube	2339 reflections with *I* > 2σ(*I*)
graphite	*R*_int_ = 0.068
φ and ω scans	θ_max_ = 26.0°, θ_min_ = 2.3°
Absorption correction: integration (*SADABS*; Bruker, 2009)	*h* = −12→12
*T*_min_ = 0.545, *T*_max_ = 0.909	*k* = −9→9
20490 measured reflections	*l* = −41→43

### Refinement

**Table:**

Refinement on *F*^2^	Primary atom site location: structure-invariant direct methods
Least-squares matrix: full	Secondary atom site location: difference Fourier map
*R*[*F*^2^ > 2σ(*F*^2^)] = 0.075	Hydrogen site location: inferred from neighbouring sites
*wR*(*F*^2^) = 0.153	H-atom parameters constrained
*S* = 1.26	*w* = 1/[σ^2^(*F*_o_^2^) + (0.*P*)^2^ + 19.462*P*] where *P* = (*F*_o_^2^ + 2*F*_c_^2^)/3
2752 reflections	(Δ/σ)_max_ = 0.004
172 parameters	Δρ_max_ = 0.49 e Å^−^^3^
0 restraints	Δρ_min_ = −0.75 e Å^−^^3^

### Special details

**Table:**

Geometry. All e.s.d.'s (except the e.s.d. in the dihedral angle between two l.s. planes) are estimated using the full covariance matrix. The cell e.s.d.'s are taken into account individually in the estimation of e.s.d.'s in distances, angles and torsion angles; correlations between e.s.d.'s in cell parameters are only used when they are defined by crystal symmetry. An approximate (isotropic) treatment of cell e.s.d.'s is used for estimating e.s.d.'s involving l.s. planes.
Refinement. Refinement of *F*^2^ against ALL reflections. The weighted *R*-factor *wR* and goodness of fit *S* are based on *F*^2^, conventional *R*-factors *R* are based on *F*, with *F* set to zero for negative *F*^2^. The threshold expression of *F*^2^ > σ(*F*^2^) is used only for calculating *R*-factors(gt) *etc*. and is not relevant to the choice of reflections for refinement. *R*-factors based on *F*^2^ are statistically about twice as large as those based on *F*, and *R*- factors based on ALL data will be even larger.

### Fractional atomic coordinates and isotropic or equivalent isotropic displacement parameters (Å^2^)

**Table:**

	*x*	*y*	*z*	*U*_iso_*/*U*_eq_	
C1	0.5039 (8)	0.7838 (8)	0.40483 (18)	0.0425 (17)	
H1	0.4331	0.8509	0.3961	0.051*	
C2	0.5565 (8)	0.7851 (9)	0.44168 (19)	0.0459 (19)	
H2	0.5266	0.8551	0.4620	0.055*	
C3	0.6571 (7)	0.6697 (10)	0.4435 (2)	0.0461 (18)	
H3	0.7090	0.6465	0.4650	0.055*	
C4	0.6699 (7)	0.5910 (9)	0.4073 (2)	0.0430 (17)	
H4	0.7308	0.5040	0.4004	0.052*	
C5	0.5767 (7)	0.6644 (7)	0.38373 (17)	0.0348 (15)	
H5	0.5648	0.6379	0.3578	0.042*	
C6	0.5240 (6)	0.3405 (8)	0.45376 (15)	0.0279 (13)	
C7	0.3395 (7)	0.5626 (8)	0.45266 (17)	0.0366 (15)	
C8	0.3943 (6)	0.3817 (8)	0.39055 (16)	0.0333 (14)	
H8A	0.3294	0.3098	0.4039	0.040*	
H8B	0.3476	0.4590	0.3728	0.040*	
C9	0.4800 (6)	0.2613 (8)	0.36798 (16)	0.0327 (14)	
H9A	0.5282	0.1825	0.3851	0.039*	
H9B	0.5424	0.3302	0.3530	0.039*	
C10	0.4778 (7)	−0.0152 (8)	0.31408 (16)	0.0377 (15)	
C11	0.6121 (7)	−0.0106 (7)	0.31470 (16)	0.0357 (15)	
H11	0.6553	0.0756	0.3293	0.043*	
C12	0.6823 (7)	−0.1315 (9)	0.29414 (17)	0.0392 (16)	
H12	0.7736	−0.1261	0.2943	0.047*	
C13	0.6207 (8)	−0.2602 (9)	0.27334 (18)	0.0471 (19)	
H13	0.6691	−0.3431	0.2593	0.056*	
C14	0.4893 (9)	−0.2665 (10)	0.27328 (18)	0.053 (2)	
H14	0.4468	−0.3551	0.2592	0.063*	
C15	0.4172 (8)	−0.1468 (9)	0.29330 (17)	0.0453 (18)	
H15	0.3260	−0.1538	0.2930	0.054*	
Fe1	0.48739 (8)	0.53522 (10)	0.42952 (2)	0.0264 (2)	
O1	0.5463 (4)	0.2142 (5)	0.46915 (12)	0.0375 (11)	
O2	0.2434 (5)	0.5784 (7)	0.46790 (14)	0.0558 (14)	
S1	0.37701 (18)	0.1351 (2)	0.33694 (5)	0.0407 (4)	

### Atomic displacement parameters (Å^2^)

**Table:**

	*U*^11^	*U*^22^	*U*^33^	*U*^12^	*U*^13^	*U*^23^
C1	0.059 (5)	0.025 (3)	0.043 (4)	−0.004 (3)	0.005 (4)	0.014 (3)
C2	0.066 (5)	0.035 (4)	0.037 (4)	−0.024 (4)	0.015 (3)	0.002 (3)
C3	0.043 (4)	0.050 (4)	0.045 (4)	−0.022 (4)	−0.010 (3)	0.017 (3)
C4	0.034 (4)	0.039 (4)	0.055 (4)	−0.002 (3)	0.012 (3)	0.018 (3)
C5	0.052 (4)	0.025 (3)	0.028 (3)	−0.008 (3)	0.008 (3)	0.006 (2)
C6	0.026 (3)	0.033 (3)	0.025 (3)	0.003 (3)	0.000 (2)	0.000 (2)
C7	0.046 (4)	0.033 (3)	0.031 (3)	0.004 (3)	−0.001 (3)	0.003 (3)
C8	0.035 (4)	0.032 (3)	0.034 (3)	−0.005 (3)	−0.003 (3)	0.006 (3)
C9	0.041 (4)	0.027 (3)	0.031 (3)	−0.006 (3)	0.001 (3)	−0.002 (2)
C10	0.058 (5)	0.033 (3)	0.022 (3)	−0.011 (3)	−0.008 (3)	0.006 (2)
C11	0.058 (4)	0.020 (3)	0.030 (3)	−0.002 (3)	−0.004 (3)	0.004 (2)
C12	0.049 (4)	0.036 (3)	0.033 (3)	0.002 (3)	0.003 (3)	0.008 (3)
C13	0.078 (6)	0.031 (3)	0.033 (4)	0.003 (4)	0.003 (4)	0.001 (3)
C14	0.079 (6)	0.047 (4)	0.033 (3)	−0.016 (4)	−0.002 (4)	−0.010 (3)
C15	0.061 (5)	0.042 (4)	0.032 (3)	−0.014 (4)	−0.009 (3)	0.003 (3)
Fe1	0.0295 (5)	0.0238 (4)	0.0259 (4)	−0.0012 (4)	0.0019 (4)	0.0043 (3)
O1	0.043 (3)	0.030 (2)	0.040 (2)	0.001 (2)	0.001 (2)	0.007 (2)
O2	0.044 (3)	0.063 (4)	0.060 (3)	0.009 (3)	0.016 (3)	0.006 (3)
S1	0.0453 (11)	0.0401 (9)	0.0367 (8)	−0.0059 (8)	−0.0100 (8)	−0.0045 (7)

### Geometric parameters (Å, °)

**Table:**

C1—C5	1.401 (9)	C8—C9	1.510 (8)
C1—C2	1.415 (9)	C8—Fe1	2.054 (6)
C1—Fe1	2.098 (6)	C8—H8A	0.9900
C1—H1	0.9489	C8—H8B	0.9900
C2—C3	1.370 (11)	C9—S1	1.812 (6)
C2—Fe1	2.085 (6)	C9—H9A	0.9900
C2—H2	0.9490	C9—H9B	0.9900
C3—C4	1.422 (10)	C10—C15	1.396 (9)
C3—Fe1	2.101 (7)	C10—C11	1.397 (10)
C3—H3	0.9500	C10—S1	1.753 (7)
C4—C5	1.396 (9)	C11—C12	1.384 (9)
C4—Fe1	2.098 (7)	C11—H11	0.9500
C4—H4	0.9501	C12—C13	1.386 (9)
C5—Fe1	2.114 (6)	C12—H12	0.9500
C5—H5	0.9485	C13—C14	1.367 (11)
C6—O1	1.132 (7)	C13—H13	0.9500
C6—Fe1	1.759 (6)	C14—C15	1.379 (10)
C7—O2	1.142 (8)	C14—H14	0.9500
C7—Fe1	1.755 (7)	C15—H15	0.9500
			
C5—C1—C2	106.7 (6)	C11—C10—S1	125.1 (5)
C5—C1—Fe1	71.2 (3)	C12—C11—C10	120.2 (6)
C2—C1—Fe1	69.7 (4)	C12—C11—H11	119.9
C5—C1—H1	126.7	C10—C11—H11	119.9
C2—C1—H1	126.6	C11—C12—C13	120.6 (7)
Fe1—C1—H1	124.1	C11—C12—H12	119.7
C3—C2—C1	109.5 (7)	C13—C12—H12	119.7
C3—C2—Fe1	71.5 (4)	C14—C13—C12	119.2 (7)
C1—C2—Fe1	70.7 (4)	C14—C13—H13	120.4
C3—C2—H2	125.3	C12—C13—H13	120.4
C1—C2—H2	125.2	C13—C14—C15	121.3 (7)
Fe1—C2—H2	124.0	C13—C14—H14	119.4
C2—C3—C4	107.5 (6)	C15—C14—H14	119.4
C2—C3—Fe1	70.3 (4)	C14—C15—C10	120.2 (7)
C4—C3—Fe1	70.1 (4)	C14—C15—H15	119.9
C2—C3—H3	126.3	C10—C15—H15	119.9
C4—C3—H3	126.2	C7—Fe1—C6	93.6 (3)
Fe1—C3—H3	125.1	C7—Fe1—C8	88.2 (3)
C5—C4—C3	107.7 (6)	C6—Fe1—C8	87.0 (3)
C5—C4—Fe1	71.2 (4)	C7—Fe1—C2	95.5 (3)
C3—C4—Fe1	70.3 (4)	C6—Fe1—C2	126.8 (3)
C5—C4—H4	126.1	C8—Fe1—C2	145.5 (3)
C3—C4—H4	126.2	C7—Fe1—C4	160.7 (3)
Fe1—C4—H4	123.9	C6—Fe1—C4	99.1 (3)
C4—C5—C1	108.5 (6)	C8—Fe1—C4	106.9 (3)
C4—C5—Fe1	70.0 (3)	C2—Fe1—C4	65.2 (3)
C1—C5—Fe1	70.0 (3)	C7—Fe1—C1	99.1 (3)
C4—C5—H5	125.7	C6—Fe1—C1	162.0 (3)
C1—C5—H5	125.8	C8—Fe1—C1	106.0 (3)
Fe1—C5—H5	125.9	C2—Fe1—C1	39.6 (3)
O1—C6—Fe1	179.2 (6)	C4—Fe1—C1	65.5 (3)
O2—C7—Fe1	179.2 (6)	C7—Fe1—C3	124.6 (3)
C9—C8—Fe1	115.2 (4)	C6—Fe1—C3	96.7 (3)
C9—C8—H8A	108.5	C8—Fe1—C3	146.4 (3)
Fe1—C8—H8A	108.5	C2—Fe1—C3	38.2 (3)
C9—C8—H8B	108.5	C4—Fe1—C3	39.6 (3)
Fe1—C8—H8B	108.5	C1—Fe1—C3	65.6 (3)
H8A—C8—H8B	107.5	C7—Fe1—C5	133.5 (3)
C8—C9—S1	107.3 (4)	C6—Fe1—C5	132.4 (3)
C8—C9—H9A	110.3	C8—Fe1—C5	87.6 (2)
S1—C9—H9A	110.3	C2—Fe1—C5	65.1 (2)
C8—C9—H9B	110.3	C4—Fe1—C5	38.7 (3)
S1—C9—H9B	110.3	C1—Fe1—C5	38.9 (2)
H9A—C9—H9B	108.5	C3—Fe1—C5	65.4 (3)
C15—C10—C11	118.5 (7)	C10—S1—C9	106.0 (3)
C15—C10—S1	116.4 (6)		
			
C5—C1—C2—C3	−0.6 (7)	C5—C4—Fe1—C8	63.4 (4)
Fe1—C1—C2—C3	61.4 (5)	C3—C4—Fe1—C8	−179.1 (4)
C5—C1—C2—Fe1	−62.0 (4)	C5—C4—Fe1—C2	−80.6 (4)
C1—C2—C3—C4	−0.4 (7)	C3—C4—Fe1—C2	37.0 (4)
Fe1—C2—C3—C4	60.5 (4)	C5—C4—Fe1—C1	−36.9 (4)
C1—C2—C3—Fe1	−60.8 (5)	C3—C4—Fe1—C1	80.7 (4)
C2—C3—C4—C5	1.2 (7)	C5—C4—Fe1—C3	−117.6 (6)
Fe1—C3—C4—C5	61.8 (4)	C3—C4—Fe1—C5	117.6 (6)
C2—C3—C4—Fe1	−60.6 (5)	C5—C1—Fe1—C7	−155.6 (4)
C3—C4—C5—C1	−1.6 (7)	C2—C1—Fe1—C7	87.6 (5)
Fe1—C4—C5—C1	59.6 (4)	C5—C1—Fe1—C6	69.9 (9)
C3—C4—C5—Fe1	−61.2 (4)	C2—C1—Fe1—C6	−46.9 (10)
C2—C1—C5—C4	1.4 (7)	C5—C1—Fe1—C8	−64.9 (5)
Fe1—C1—C5—C4	−59.6 (4)	C2—C1—Fe1—C8	178.4 (4)
C2—C1—C5—Fe1	61.0 (4)	C5—C1—Fe1—C2	116.7 (6)
Fe1—C8—C9—S1	178.1 (3)	C5—C1—Fe1—C4	36.8 (4)
C15—C10—C11—C12	1.9 (9)	C2—C1—Fe1—C4	−80.0 (5)
S1—C10—C11—C12	−177.0 (4)	C5—C1—Fe1—C3	80.4 (5)
C10—C11—C12—C13	−1.3 (9)	C2—C1—Fe1—C3	−36.3 (4)
C11—C12—C13—C14	0.1 (10)	C2—C1—Fe1—C5	−116.7 (6)
C12—C13—C14—C15	0.4 (11)	C2—C3—Fe1—C7	−46.6 (5)
C13—C14—C15—C10	0.3 (11)	C4—C3—Fe1—C7	−164.7 (4)
C11—C10—C15—C14	−1.4 (9)	C2—C3—Fe1—C6	−145.7 (4)
S1—C10—C15—C14	177.6 (5)	C4—C3—Fe1—C6	96.2 (4)
C9—C8—Fe1—C7	−160.1 (5)	C2—C3—Fe1—C8	119.7 (5)
C9—C8—Fe1—C6	−66.4 (5)	C4—C3—Fe1—C8	1.6 (7)
C9—C8—Fe1—C2	102.8 (6)	C4—C3—Fe1—C2	−118.1 (6)
C9—C8—Fe1—C4	32.3 (5)	C2—C3—Fe1—C4	118.1 (6)
C9—C8—Fe1—C1	100.9 (5)	C2—C3—Fe1—C1	37.6 (4)
C9—C8—Fe1—C3	31.2 (7)	C4—C3—Fe1—C1	−80.5 (4)
C9—C8—Fe1—C5	66.3 (4)	C2—C3—Fe1—C5	80.5 (4)
C3—C2—Fe1—C7	143.1 (4)	C4—C3—Fe1—C5	−37.6 (4)
C1—C2—Fe1—C7	−97.6 (5)	C4—C5—Fe1—C7	153.7 (5)
C3—C2—Fe1—C6	44.4 (5)	C1—C5—Fe1—C7	34.2 (6)
C1—C2—Fe1—C6	163.7 (4)	C4—C5—Fe1—C6	−37.4 (5)
C3—C2—Fe1—C8	−122.0 (6)	C1—C5—Fe1—C6	−156.9 (4)
C1—C2—Fe1—C8	−2.7 (8)	C4—C5—Fe1—C8	−121.1 (4)
C3—C2—Fe1—C4	−38.3 (4)	C1—C5—Fe1—C8	119.4 (4)
C1—C2—Fe1—C4	81.0 (5)	C4—C5—Fe1—C2	80.7 (5)
C3—C2—Fe1—C1	−119.3 (6)	C1—C5—Fe1—C2	−38.8 (4)
C1—C2—Fe1—C3	119.3 (6)	C1—C5—Fe1—C4	−119.5 (6)
C3—C2—Fe1—C5	−81.1 (4)	C4—C5—Fe1—C1	119.5 (6)
C1—C2—Fe1—C5	38.1 (4)	C4—C5—Fe1—C3	38.4 (4)
C5—C4—Fe1—C7	−76.5 (10)	C1—C5—Fe1—C3	−81.0 (5)
C3—C4—Fe1—C7	41.1 (10)	C15—C10—S1—C9	169.7 (5)
C5—C4—Fe1—C6	153.0 (4)	C11—C10—S1—C9	−11.5 (6)
C3—C4—Fe1—C6	−89.5 (4)	C8—C9—S1—C10	−175.3 (4)
